# Molecular Mechanisms Underlying Cancer Prevention and Intervention with Bioactive Food Components

**DOI:** 10.3390/cancers15133383

**Published:** 2023-06-28

**Authors:** Anupam Bishayee

**Affiliations:** College of Osteopathic Medicine, Lake Erie College of Osteopathic Medicine, Bradenton, FL 34211, USA; abishayee@lecom.edu or abishayee@gmail.com

Cancer is the second-leading cause of death in the world, and it represents a major health challenge. According to the International Agency for Research on Cancer/GLOBOCAN, more than 19 million new cancer cases and almost 10 million cancer deaths occurred in the year 2020 [1]. The overwhelming evidence, which is based on preclinical and clinical studies, clearly indicates that diet can modify cancer outcome. Natural dietary bioactive compounds, present in fruits, vegetables, spices, whole grains, and herbs, have shown enormous potential for cancer prevention and treatment due to their easy availability, relatively low cost, high margin of safety, widespread acceptability, and human consumption.

During the last few decades, an extraordinary number of bioactive food components have been investigated, employing cell culture assays, animal tumor models, and human subjects to understand their potential for cancer prevention and treatment. I am pleased to introduce this Special Issue, which captures recent advances in our knowledge on cancer preventive and therapeutic efficacy of putative food-derived substances with understanding of the underlying cellular and molecular mechanisms of action. This thematic issue contains five original research papers and eight review articles.

Phannasorn and coinvestigators [2] evaluated the chemopreventive effects of riceberry bran oil, containing phytosterols, γ-oryzanol, and γ-tocotrienol, in chemically induced liver and colon carcinogenesis in rats. Oral administration of riceberry bran oil suppressed preneoplastic hepatic lesions and colorectal aberrant crypt foci, induced hepatocellular and colorectal cell apoptosis, and reduced the expression of proinflammatory cytokines. Additionally, the oil promoted the alteration of gut microbiota in both tumor models. This outcome of these experimental results indicates the potential health benefits of the consumption of rice constituents in preventing hepatic and colorectal cancers.

β-Caryophyllene is the primary sesquiterpene present in black pepper, cloves, hops, rosemary, copaiba, and cannabis. It has been recognized as the first known “dietary cannabinoid,” a common component of food with a “Generally Recognized as Safe” status. It is approved by the United States Food and Drug Administration as a taste enhancer, food additive, and flavoring agent. According to a study conducted by Mannino and colleagues [3], β-caryophyllene has been found to suppress cell proliferation and to induce apoptosis by impacting the crosstalk between Akt, β-catenin, and cyclin D/cyclin-dependent kinase 4/6 signaling in a concentration-dependent manner in multiple myeloma. These results indicate that β-caryophyllene may represent an interesting alternative or additional therapeutic option to conventional chemotherapy for the treatment of multiple myeloma.

Polygodial, a natural sesquiterpene, can be extracted from water pepper (*Persicaria hydropiper*), Dorrigo pepper (*Tasmannia stipitata*), and mountain pepper (*Tasmannia lanceolata*). The results of a study conducted by Venkatesan et al. [4] showed that polygodial effectively inhibited the viability, cell cycle progression, and migration of taxane-resistant prostate cancer cells, possibly by increasing the generation of reactive oxygen species, disrupting the mitochondrial membrane, and activating the intrinsic cell death pathway.

Parupathi et al. [5] investigated the potential of gnetin C, a phytocompound found in the melinjo plant and commonly used in Indonesian foods, to block prostate cancer progression. Their findings demonstrate that a gnetin C-supplemented diet effectively suppresses metastasis-associated protein 1-promoted tumor progression in high-risk premalignant prostate cancer transgenic mouse model. This study underscores the potential of gnetin C as a novel nutritional agent for prostate cancer prevention.

The study by Raina et al. [6] focused on elucidating the “stage-specific” efficacy of the bioactive food component inositol hexaphosphate (IP6, also known as phytic acid) against prostate cancer initiation, growth, and progression in a transgenic adenocarcinoma of the mouse prostate (TRAMP) model. Results indicated that IP6 feeding during the initial stages of cancer development prevents the progression of prostatic intraepithelial neoplasia lesions to adenocarcinoma, and IP6 feeding during the late stage of the disease reduces tumor growth and prevents its progression to the advanced stage of the disease. It has also been indicated that the anti-prostate cancer effects of IP6 are associated with its potential to eradicate the prostate cancer stem cell pool in the TRAMP model. Accordingly, IP6 intervention could have a therapeutic benefit during all stages of prostate tumorigenesis.

In this Special Issue, three review articles capture recent developments regarding research, elucidating the role of dietary phytochemicals on gastrointestinal tract cancers. Kang et al. [7] summarized the potential therapeutic effects of bioactive food components on the prevention and treatment of gastric cancer, with special focus on molecular mechanisms of action, bioavailability, and safety aspects. De et al. [8] reviewed the literature on phenolic phytocompounds endowed with anti-colorectal cancer activities, which are based on animal and human studies, to understand the impact of these results on the prevention and treatment of this cancer, which represents a significant cause of death worldwide. Dacrema et al. [9] presented an overview of the reciprocal interactions between spice-derived bioactive compounds and the gut microbiota to understand the role of dietary spices in the prevention of colorectal cancer.

There are two reviews dedicated to the impact of food-derived phytochemicals on hormone-related neoplasms. Prajapati and colleagues [10] dissected the concept of targeting luminal A-derived breast cancer stem cells with dietary phytocompounds by summarizing the signaling pathways implicated in therapy resistance. In their review, Kumar et al. [11] discussed the role of green tea catechins in the prevention of prostate cancer, presented evidence on the associations of microbiomes with prostate cancer, and evaluated the concept of utilizing the microbiome to identify biomarkers for the efficacy of green tea-derived constituents.

*Nelumbo nucifera* Gaertn., also known as the lotus, sacred lotus, Indian lotus, or Chinese water lily, is a recognized dietary and medicinal plant. Bishayee and colleagues [12] critically evaluated the potential of *N. nucifera*-derived products and phytoconstituents in cancer prevention and intervention with in-depth understanding of cellular and molecular mechanisms of action. Sulforaphane represents a metabolite of the phytochemical glucoraphanin, which is present in cruciferous vegetables, such as broccoli, Brussels sprouts, cabbage, and watercress. A review by Kaiser et al. [13] evaluated the recent state of knowledge on the efficacy of sulforaphane in preventing or reversing a variety of neoplasms based on preclinical and clinical studies. The authors also discussed the current limitations and challenges associated with sulforaphane research, and they suggested future research directions.

Finally, the work of Bouyahya et al. [14] focuses on recent advances in using dietary phenolic phytocompounds to sensitize various cancer cells towards chemotherapeutic agents and their values, in combination therapy, along with conventional anticancer drugs. Several phenolics, including caffeic acid, curcumin, gallic acid, resveratrol, rosmarinic acid, and sinapic acid, exhibit encouraging anticancer activities through sub-cellular, cellular, and molecular mechanisms, and they can increase the effectiveness of the approved cancer chemotherapeutic agents.

In conclusion, it is my hope that this Special Issue, featuring high-quality articles written by recognized leaders in the field, as well as young investigators from all over the world, would accelerate the translational impact of mechanism-based cancer prevention and intervention using multi-targeted dietary phytocompounds, identify current knowledge gaps, challenges, and pitfalls, as well as galvanize future research.
